# Supplementary material for: Type I interferon is required for T helper (Th) 2 induction by dendritic cells
Source: EMBO J. 2017 Jul 17;36(16):2404–18. doi: 10.15252/embj.201695345 (PMC5556270; doi:10.15252/embj.201695345)
Supplement: Supplementary file 1 — Expanded View Figures PDF [file EMBJ-36-2404-s001.pdf]

Expanded View Figures

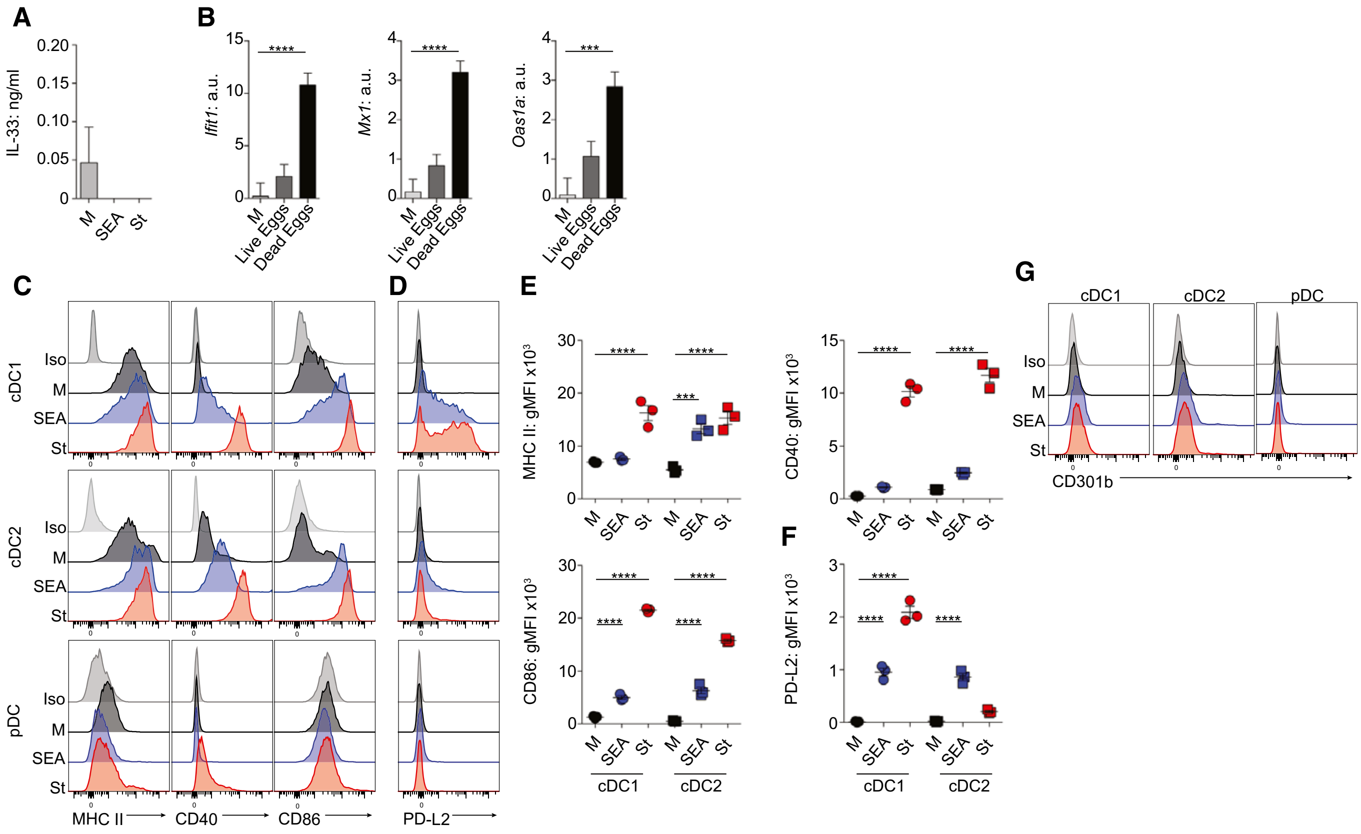

Figure EV1. FLDCs' responses to Ag.

A IL-33 production by FLDCs cultured in medium alone (M) or with Ag.
B WT FLDCs were cultured at a ratio of 1 live or dead egg: 500 DCs, or in medium alone (M), for 6 h for gene expression analysis (normalized against *Gapdh*, a.u.).
C, D DC phenotype following overnight culture in medium alone (M, black), SEA (blue), or St (red). Isotype control (Iso, gray-shaded).
E, F gMFI of surface markers for cDC1 and cDC2 FLDC subsets.
G CD301b expression of FLDC subsets following 18-h culture in the presence or absence of Ag.

Data information: Results are mean ± SEM (A, E, F) (one-way ANOVA) or least squares mean ± SEM (B) (analyzed using a three-way full-factorial fit model, with contrast analysis used to test differences between experimental groups). ***$P < 0.001$, ****$P < 0.0001$. Data from one of three or more experiments ($n = 3$ replicate wells per group) (A, C–G) or three experiments pooled ($n = 7$–8 replicate wells) (B). gMFI, geometric mean fluorescence intensity. a.u., arbitrary units.

 © 2017 The Authors

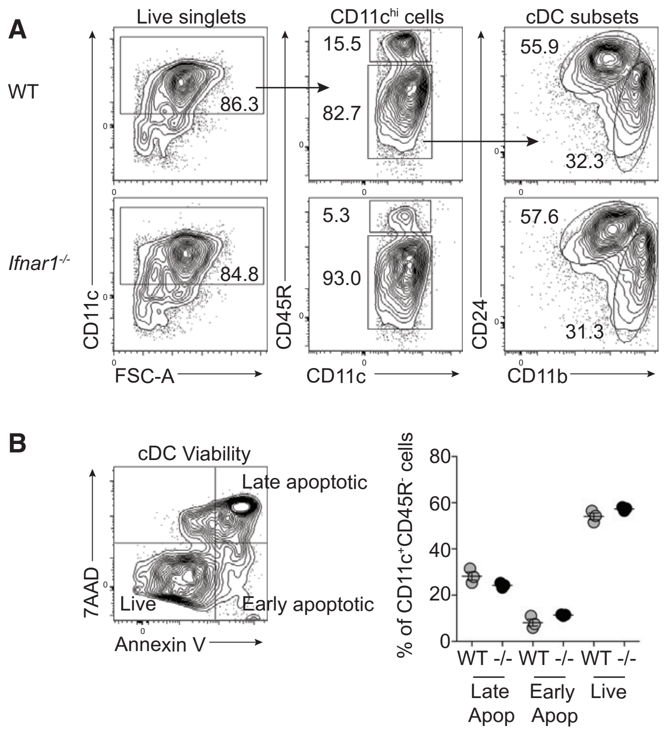

Figure EV2. *Ifnar1*$^{-/-}$ FLDCs display normal development and viability.

A FLDCs were cultured for 18 h in medium alone and analyzed for subsets by flow cytometry.

B Following 18-h culture, FLDCs were stained with 7AAD and Annexin V to assess viability of cDC populations. 7AAD^{+} Annexin V^{+} were classed as late apoptotic, 7AAD^{-} Annexin V^{+} as early apoptotic, and 7AAD^{-} Annexin V^{-} as live cells. Results are mean ± SEM.

Data information: Data from one of three or more experiments (n = 3 replicate wells per group).

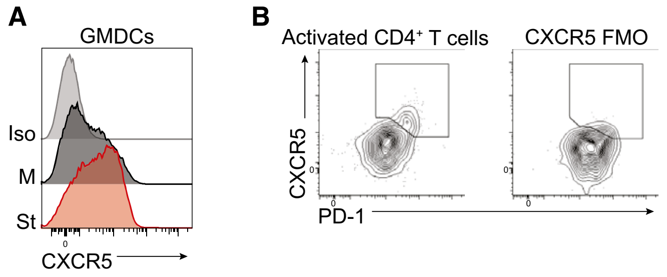

Figure EV3. St elicits a strong upregulation of CXCR5 on the surface of GMDCs.

A GMDCs were cultured for 18 h in medium alone (M) or in the presence of St, and the expression of CXCR5 was analyzed by flow cytometry and compared to an isotype control (Iso).

B Expression of CXCR5 and PD-1 on activated (CD44^{hi} CD62L^{lo}) CD4^{+} Foxp3^{-} T cells from hepatic LN of *Schistosoma mansoni*-infected mouse on day 42 of infection. FMO (fluorescence minus one) of CXCR5 expression.

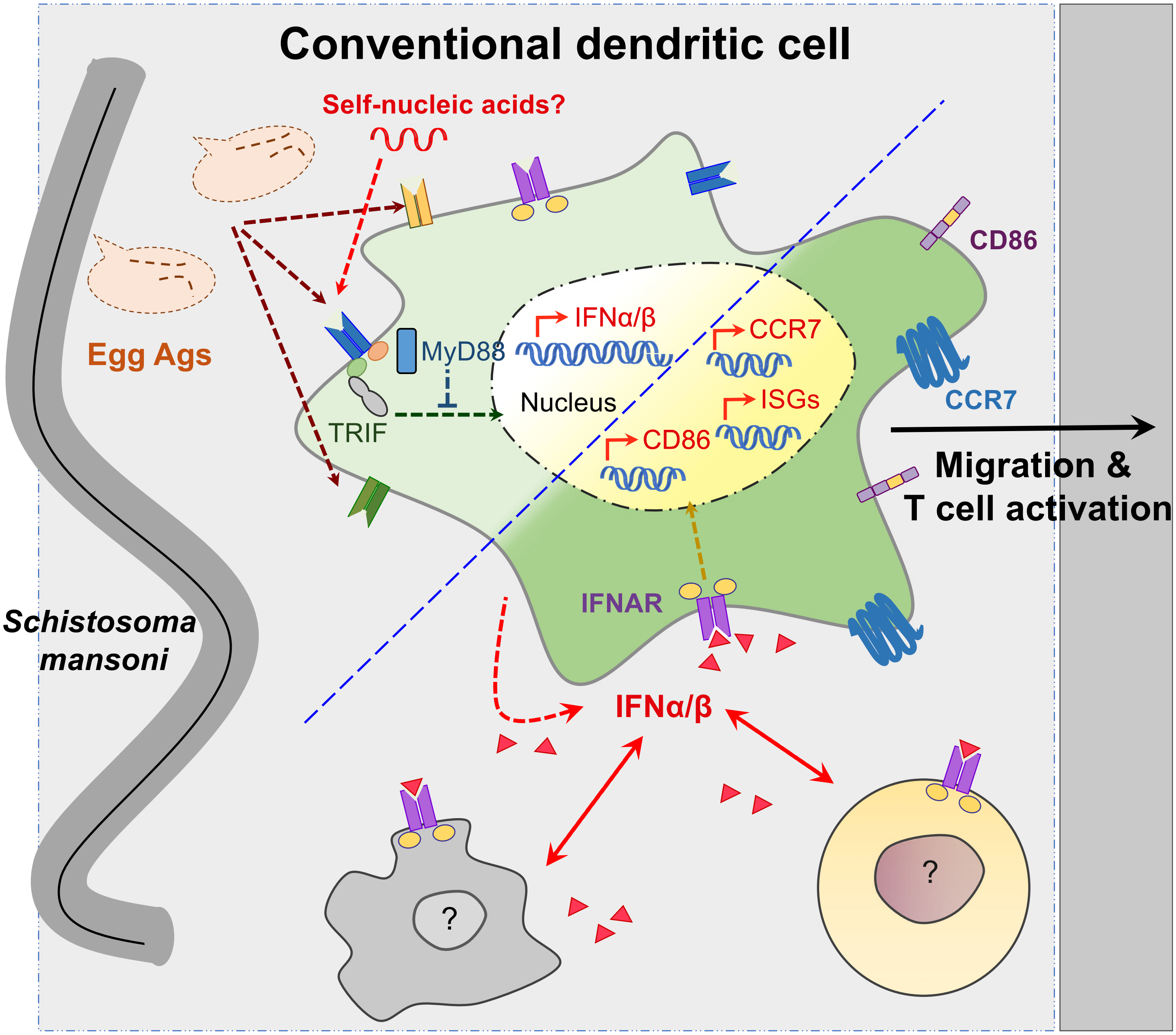

Figure EV4. A TRIF-dependent IFN-I signature is induced in response to *Schistosoma mansoni* egg Ags and is required for optimal DC activation, migration, and Th2 priming capacity.

Th2-inducing Ags, such as those released from *S. mansoni* eggs, bind to a range of Ag receptors on the surface of cDCs. Ags such as omega-1, which binds the mannose receptor, likely act in concert with parasite- and/or host-derived nucleic acid ligands that bind TRIF-dependent receptors and activate the transcription of IFN-I genes. This leads to the secretion of IFN-I, including IFNα subtypes and IFNβ. cDCs themselves express the IFN-I receptor IFNAR and can therefore respond to IFN-I produced from a range of sources including DCs and other immune cells. This stimulates the activation of IFN-dependent signaling pathways which leads to transcription of a range of IFN-dependent genes, including ISGs, and upregulation of surface markers such as CCR7 and CD86. In this way, IFN-I signaling enhances the activation, migration, and antigen presentation capabilities of cDCs during type 2 inflammation.

 © 2017 The Authors
